# The impact of smartphone use on rural residents’ environmental quality assessment: evidence from micro-level survey data in China

**DOI:** 10.3389/fpubh.2025.1705375

**Published:** 2026-01-09

**Authors:** Fan Chen, Yuhuan Chen, Sheng Shi

**Affiliations:** 1 China Rural Policy and Practice Research Institute, Ningbo University, Ningbo, China; 2 Zhejiang Provincial Research Think Tank Alliance for Rural Revitalization, Ningbo, China; 3 Business School of Ningbo University, Ningbo, China

**Keywords:** digitalization, environmental quality assessment, environmental satisfaction, mechanism analysis, smartphone usage

## Abstract

**Background:**

The popularization of smartphones and the development of internet digitalization are profoundly reshaping the thinking patterns and behavioral choices of rural residents, while also exerting a significant impact on their environmental quality perception and wellbeing. However, existing studies have not fully clarified the interactive relationship between smartphone use and residents’ environmental cognition, which provides an important entry point for this research.

**Methods:**

Based on micro-survey data from rural China, this study employs an empirical approach to examine the impact of smartphone use on rural residents’ assessments of environmental quality, and innovatively explores its inherent mechanism of action.

**Conclusion and discussion:**

The research finds that smartphone use has a significant negative impact on rural residents’ satisfaction with environmental quality, and this conclusion remains valid after multiple robustness tests. Heterogeneity analysis shows that this impact exhibits obvious regional and group differences: compared with the central region, the negative effect is more moderate in the eastern and western regions; in contrast to ordinary villagers, the impact of smartphone use on the environmental quality satisfaction of rural Party members and cadres is not significant. Mechanism analysis reveals that smartphone use affects environmental satisfaction by changing rural residents’ information acquisition channels and promoting the transformation of social capital forms. This study provides empirical evidence and theoretical reference for the rational use of digital tools to guide residents’ environmental perception and support the construction of ecologically livable rural areas.

## 1 Introduction

Globally, rural environmental governance has become a critical component of sustainable development (1). The United Nations Sustainable Development Goal (SDG) 11 explicitly calls for “making cities and human settlements inclusive, safe, resilient and sustainable”—a target that also encompasses rural areas and emphasizes the integration of environmental quality with residents’ wellbeing. The World Bank’s *Poverty and Shared Prosperity 2023* report indicates that in low- and middle-income countries, approximately 34% of the rural population (equivalent to around 1.2 billion people) is experiencing severe challenges such as lack of improved sanitation facilities, water pollution, waterborne diseases, and unsustainable resource use. These issues not only cause objective harm to health and livelihoods but also shape negative perceptions of environmental quality among residents. Similarly, studies have shown that residents’ perceptions of environmental quality in developed countries also influence rural population retention and community cohesion (2, 33). As a subjective perception rooted in residents, daily life experiences, environmental quality assessment is not merely a regional issue but a crucial matter related to rural sustainable development.

China also attaches great importance to subjective perceptions of environmental quality. The 2025 China Central Government’s “No.1Document” emphasizes “sustainably promoting the improvement of rural living environments,” fully recognizing that objective environmental improvements must align with residents’ subjective satisfaction to achieve lasting effectiveness. Over the past four decades of reform and opening up, China’s rural environmental governance has shifted from lax management to systematic governance, yet challenges such as pollution and unsustainable resource use persist (3, 4). Notably, these objective environmental problems are not directly equivalent to residents’ subjective evaluations; on the contrary, how rural residents perceive and assess their living environments plays a decisive role in their acceptance of governance measures and the enhancement of their overall wellbeing (5, 6, 33). China’s “Thousand-Village Demonstration and Ten-Thousand-Village Renovation” program also emphasizes promoting “informatization of rural living environment governance,” highlighting the connection between digitalization and subjective perceptions, and recognizing that digital tools can influence how residents access environmental information and form evaluations. Against this dual global and national backdrop, rural residents’ environmental quality assessment, as a core form of subjective environmental awareness, has become a key indicator for measuring the effectiveness of rural living environment construction. Their perceptions directly impact behavioral responses to environmental policies, participation in rural governance, and the outcomes of rural revitalization initiatives. With the popularization of smartphones among rural Chinese residents, rural adults now own smartphones and use them for information retrieval, social interaction, and participation in public affairs. Exploring how smartphone use shapes these subjective perceptions is both timely and of great significance.

Existing studies have shown that the use of digital tools such as smartphones can broaden the channels for environmental information dissemination and play a positive role in popularizing knowledge about environmental issues such as pollution and smog. It not only conveys environmental awareness to rural residents and improves their level of environmental risk perception but also indirectly shapes positive environmental perceptions by enhancing individuals’ self-efficacy in environmental behaviors (7–9). For instance, based on survey data from rural China, Liu et al. (9) found that the digital economy has significantly optimized the environmental cognitive structure of rural residents through information empowerment and resource integration, providing cognitive support for the improvement of rural living environments. Zheng et al. (33) out in their research that among groups with strong information screening capabilities, smartphone use can instead improve their environmental satisfaction by accurately transmitting information on environmental governance outcomes. Through an analysis of nationwide survey data, Yi et al. (10) discovered that internet use (including smartphones) can significantly and positively influence the comprehensiveness and objectivity of the public’s environmental pollution perception by enhancing their environmental knowledge reserve and participation awareness, without showing obvious negative effects. Chen and Ye (11) focused more on the improvement of governance efficiency and the optimization of information access channels. However, the widespread use of smartphones may also bring negative effects: farmers may be exposed to excessive fragmented negative environmental information, thereby forming negative environmental perceptions, and such negative perceptions may be further amplified through interpersonal communication on social networks (11–14). An empirical study on rural China by Deng et al. (12, 13) confirmed that although internet use can improve farmers’ sensitivity to environmental pollution, the influx of excessive negative information reduces their evaluation of local environmental quality. Based on micro-survey data from China, Zhang et al. (15, 16) also found that internet use (with smartphones as the main carrier) has a significant negative impact on individuals’ environmental quality evaluations, and this impact is strengthened through information overload and comparison effects. Another study by Zhang et al. (15, 16) indicated that the higher the frequency of smartphone use, the lower residents’ satisfaction with the government’s environmental protection work, which in turn indirectly reduces overall environmental satisfaction. In addition, research by Zhang et al. (17) similarly confirmed that the widespread use of smartphones exerts a significant negative impact on public satisfaction with environmental governance by exacerbating environmental information anxiety, and further affects residents’ sense of happiness.

Nevertheless, there is a need for further exploration of the impact of smartphone use on rural residents’ environmental satisfaction, addressing certain shortcomings in existing research. First, previous research has focused more on residents’ happiness, with relatively less emphasis on studying residents’ perceptions of environmental quality. Environmental quality perception is a crucial aspect of wellbeing and represents a higher-level indicator for measuring residents’ happiness, which cannot be overlooked (18, 34). Second, existing research has primarily focused on urban residents or macro-level data, with less attention given to micro-level research involving rural farmers. Research on farmers’ environmental perception is critical, not only for addressing rural residents’ wellbeing but also as a key indicator for social equity and harmonious development. Particularly, in countries undergoing transitions, like China, the emphasis on the wellbeing of 500 million rural farmers has a positive impact on the shared prosperity of the people (19, 33). Third, existing research has yet to fully deconstruct the mechanisms through which smartphone use influences farmers’ environmental quality perception. A more in-depth analysis of how smartphone use affects residents’ environmental perception mechanisms can provide insights into the regularities of digitization and rural environmental perception, enabling the formulation of policies that are more attuned to the needs of rural farmers and facilitating the wider adoption of digital technology (20, 21).

Further examination reveals that it is essential to delve into the underlying principles. Residents’ environmental satisfaction is subject to dual constraints from both their personal experiences and comparative experiences (12, 13, 22). “Personal experience” refers to residents’ satisfaction derived from tangible sensory experiences of their immediate environment. “Comparative experience,” on the other hand, arises from comparing their own environment to other environments they become aware of through various means (23, 24). Comparative experience can have a profound influence on residents’ environmental satisfaction, especially when smartphone use significantly magnifies the effect of comparative experiences (11). In a digital context, smartphone development has expanded channels for residents’ communication, making it easier to transmit information between individuals (12, 13, 25). Previously, disparities in environmental quality were often isolated by geographic boundaries. With the development of the internet and the widespread adoption of smartphones, these barriers have been leveled, allowing people to more directly confront these disparities and intensifying the effect of comparative experiences on residents’ environmental quality satisfaction (26).

Therefore, there is a need to further explore how smartphone use influences rural residents’ environmental satisfaction, addressing the aforementioned limitations in current research. In comparison to existing studies, this paper aims to provide supplementary insights based on micro-level survey questionnaire data from Chinese farmers. It offers a fresh perspective in the following ways: First, in terms of research content, this paper confirms the negative impact of smartphone use on rural farmers’ environmental quality satisfaction, complementing existing research on the insufficiently explored influence of digitization on farmers’ environmental perception and providing a new angle for enhancing research on the wellbeing of rural residents in China. Second, in the context of heterogeneity testing, aside from studying the impact of media types and rural residents’ income levels, this paper analyzes differences in the influence of smartphone use in various regions and the differences in satisfaction based on villagers’ identities. These findings can serve as a basis for designing precise environmental governance policies. Third, in terms of theoretical mechanisms, this paper delves into the mechanisms through which smartphone use affects rural residents’ environmental quality satisfaction. By focusing on the expansion of information channels and the transformation of social capital, this paper provides an analysis of the mechanisms that existing literature has yet to address. Consequently, this research contributes to the fields of information technology economics and environmental economics and provides a foundation for governments in their efforts to better utilize new technology platforms and formulate more precise policies (26).

The structure of this paper is as follows: Section 2 discusses the analysis of real-world characteristics and theoretical mechanisms, outlining the principles by which digitization affects environmental satisfaction. Section 3 presents the research design, while Section 4 covers empirical testing. Through baseline regression, robustness analysis, heterogeneity exploration, and mechanism analysis, this paper examines the hypotheses in-depth, contributing to a comprehensive understanding of the mechanisms involved. Finally, Section 5 presents the conclusions and recommendations.

## 2 Real features and theoretical analysis

### 2.1 Real features of rural digital development

According to the *2022 China Broadband Development White Paper* published by the China Academy of Information and Communications Technology (CAICT), as of the end of November 2021, broadband networks have been extended to all 510,000 village-level units in China. The broadband access rate for both administrative villages and poverty-stricken villages has reached 100%, marking a historic resolution to the problem of poor communication in impoverished areas. In addition, According to the 56th *Statistical Report on Internet Development in China*, released by the China Internet Network Information Center (CNNIC) in July 2025, as of June 2025, the number of netizens in China reached 1.123 billion, with an internet penetration rate of 79.7%. The number of mobile internet users stood at 1.116 billion, the rural netizen base reached 322 million, and the internet penetration rate in rural areas was 69.2%.^1^

The comprehensive adoption of digital technology in rural areas has transformed the delivery of numerous public services through methods such as “Internet + Healthcare,” “Internet + Education,” and “Internet + Government Affairs.” Leveraging digital technology, rural areas now have access to low-cost, high-quality resource-sharing platforms, which have improved the efficiency of public resource allocation and promoted integrated urban and rural services.

The use of smartphones has also accelerated the efficiency of grassroots governance in rural areas, enhancing the effectiveness of various grassroots activities in politics, culture, and the environment. As of June 2022, the “Internet + Government Affairs” has enabled people to complete various tasks without leaving their homes, leading to the centralization of information from 492,000 village committees nationwide, significantly enhancing rural governance efficiency. Additionally, examples like Zhejiang’s Deqing County’s “Mogan Digital Governance Map” application demonstrate the precise and efficient results achieved in the maintenance of natural resources and grassroots governance using the “In the Palm of the Village - Deqing” application software.

Smartphone usage has also influenced the behavior and mindset of rural residents, enabling them to actively engage with political and public affairs using internet tools. Surveys have shown that the number of petitions and complaints related to environmental quality issues has been increasing year by year, particularly in rural areas. In Zhejiang Province, for example, according to the “Public Letters” section of the “People Call Me for Unified Platform” in Zhejiang Province, a total of 11,369 public letters responded to by various county-level environmental bureaus between July 20, 2017, and March 19, 2023. It is evident that, in the context of widespread internet access, rural residents’ awareness of their rights to environmental protection is on the rise, with people paying increasing attention to their local environment.

### 2.2 Theoretical analysis

Based on the Theory of Planned Behavior, individual behavior is influenced by intrinsic value perceptions, self-efficacy, and subjective norms (49, 51). In the digital era, as farmers widely use smartphones to access information online, this behavior can effectively mitigate decision-value risks arising from information asymmetry and provide a crucial information foundation for the formation of their environmental perceptions (2, 20, 27). From the perspective of the Uses and Gratifications Theory, Yi et al. (10) argue that the widespread adoption of media such as smartphones leads to competition for attention with traditional media and the selective allocation of attention. Rural residents’ access to environmental information via smartphones may enhance their environmental perceptions and gratification. Drawing on the Cluster Theory, Chen and Ye (11) suggest that industrial cluster agglomeration can reduce institutional costs and generate economies of scale. Similarly, the widespread use of smartphones significantly shortens the physical distance in information access, thereby lowering various transaction costs and substantially increasing residents’ satisfaction with environmental information acquisition.

Nevertheless, despite the existence of these positive effects, academic circles hold differing views. Based on the Negativity Bias Theory, Zhang et al. (15, 16) propose that residents tend to focus more on negative information when paying attention to environmental issues, and news media are also more adept at capturing this attention feature of residents, ultimately leading to a decrease in their satisfaction with environmental governance. This perspective has been verified by several existing studies: Yang et al. (6) and Luo et al. (28) demonstrated that the popularization of smartphones and the extensive use of the internet have a significant negative impact on public satisfaction with environmental governance. In their research exploring the influence of government image on public satisfaction with environmental governance in the context of smartphone use, Zhang et al. (29) further pointed out that the current widespread use of smartphones exerts a significant negative effect on public satisfaction with environmental governance. Indeed, negativity bias is prevalent in residents’ daily lives. The popularity of smartphones has led to asymmetry in internet information preferences, making residents more inclined to favor certain types of information. This has naturally created a market for such information, and the supply of relevant information has also increased significantly, thereby further affecting residents’ satisfaction with environmental quality (30–32). The mechanisms through which smartphone use influences rural residents’ satisfaction with environmental quality can be explored from the following two aspects:

First, the substitution effect of information channels. Positive emotions derived from a pleasant environment and effective governance can amplify individuals’ positive perceptions of the environment (3, 4, 34). However, on the other hand, negative emotions that undermine individuals’ positive environmental perceptions are more likely to spread (34). For instance, Zhang et al. (48) proposed that after encountering environmental information through the media, individuals directly confront negative emotions—such as concerns about personal threats caused by environmental pollution and destruction—thereby leading to a decline in environmental satisfaction (51). The internet acts as an “emotional amplifier,” enabling individuals’ negative emotions to radiate to the group and even the societal level through online platforms (37, 36). Replacing traditional information channels, the internet facilitates easier emotional expression for people. Compared with emotional expression in real life, individuals are more inclined to speak freely online, often at a lower cost for their remarks. This reduces the cost of emotional expression and encourages individuals to vent their dissatisfaction (52). Complaining behavior may reduce individuals’ sense of responsibility within the group, prompting people to express their dissatisfaction online to gain a sense of fulfillment, empathy, and emotional resonance (18, 37). Online emotional venting replaces irrational behaviors in real life, which may drive individuals to adopt irrational and negative perceptions in their daily lives (33, 34).

Second, the transformation effect of social capital. Rural residents may shift from a social capital structure centered on offline, individual-based interactions to a form of participation that is more inclined to be online and collective. By using smartphones and increasing their online time, they can achieve resource matching more conveniently. According to a report by QuestMobile, as of June 2025, the average daily usage duration and frequency of Chinese mobile internet users were 7.97 h and 117.9 times, respectively.^2^ Compared with the corresponding figures in 2022 (5.91 h and 87.77 times per day), this represents an increase of 2 h and 30.13 times, indicating a significant and even deeper rise in user engagement. This change has profoundly reshaped the production and lifestyle of rural residents: on one hand, it may reduce the frequency of rural residents’ participation in traditional offline activities such as local gatherings and village committee elections, affecting their original lifestyle and overall perceptions; on the other hand, the online environment contains abundant knowledge and experiences, which helps expand social resources—for example, people can access more training and educational resources related to environmental protection. The positive impact brought by equitable educational opportunities can enhance rural residents’ awareness and capabilities in participating in environmental protection and quality improvement (39). However, in terms of environmental quality satisfaction, the negative impact lies in the awakening of rights awareness, which makes people more prone to dissatisfaction with current environmental issues (40). Zhang et al. (48) proposed that even if media reports on local environmental issues are objective and impartial without emphasizing potential future risks, they will still raise public attention to the environment, and this heightened attention to environmental issues will reduce environmental quality satisfaction.

**Figure 1:**
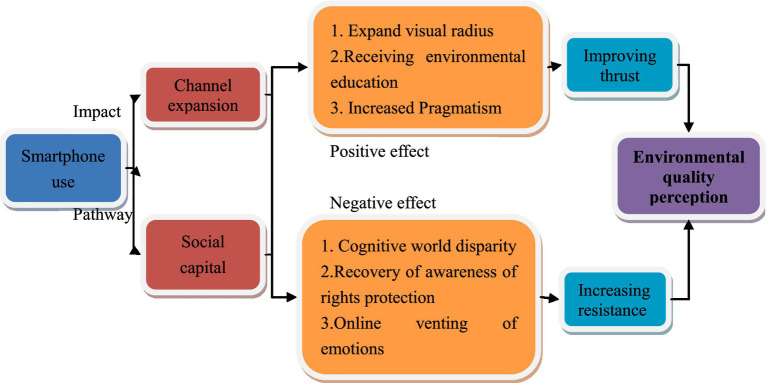
Theoretical logic of smartphones affecting farmers’ satisfaction with environmental quality.

Overall, theoretically speaking, the expansion of information channels and the transformation of social capital may lead to two distinct potential outcomes (see Figure 1): on one hand, these mechanisms can broaden horizons, enhance cognition, and improve learning abilities, which may have a positive effect on environmental quality perceptions; on the other hand, the information and abilities acquired through these channels may be insufficiently solid, leading to differences in individuals’ perceptions of the world. Coupled with the existence of emotional venting behavior, this may exert a negative impact on environmental quality satisfaction. The ultimate impact of smartphone use on environmental quality satisfaction depends on the dual role of promotion and hindrance, thereby forming differentiated effects. Nevertheless, the Negativity Bias Theory suggests that the likelihood of a negative impact is relatively high and may be more in line with realistic characteristics. Therefore, this paper proposes the following hypothesis:

*H1*: Smartphone use has a significant negative impact on residents' environmental quality perceptions.

## 3 Study design

### 3.1 Model design

To investigate the influence of smartphone use on rural residents’ satisfaction with environmental quality, this study utilizes rural residents’ satisfaction with environmental quality as the chosen explanatory variable, with the key explanatory variable being their frequency of smartphone use for acquiring knowledge. As the explanatory variables in this analysis are ordered discrete variables, it is more appropriate to employ an ordered probability model for econometric analysis, as suggested by Zhang et al. (41), and Leng et al. (36). In line with the methodology of Jiang et al. (5) this paper examines the following econometric models.


(1) $$\mathrm{Eq}s_{i}=\alpha_{0}+\alpha_{1}SU_{i}+\sum_{i=2}\alpha_{i}\text{Control}s_{i}+\upsilon_{i}$$

In the Equation 1, $\mathrm{Eq}s_{i}$ represents the satisfaction of rural residents with environmental quality, which is an ordered discrete variable with values ranging from 1 to 5. $SU_{i}$ represents Smartphone use, whether farmers frequently access smartphone, which is a binary variable with values of 0 or 1, and is the core variable of this article. $\text{Control}s_{i}$ represent other related control variables that influence EQS, such as individual samples and regional factors. ‘ $\upsilon_{i}$ ’ represents the random disturbance term.

### 3.2 Data sources

The data utilized in this study is sourced from the Rural Governance Modernization Questionnaire, which was released in 2020. The data collection process adheres to the principles of stratified sampling and employs a rigorous sampling strategy to ensure the representativeness of the sample.

Initially, the data underwent a comprehensive preliminary test. A specialized team of experts meticulously designed the initial statistical sample, which then underwent a pilot survey between February and March 2020 in select regions. Based on the feedback from the pilot survey, adjustments were made to enhance the questionnaire, ensuring its completeness and accuracy. Subsequently, to enrich the dataset, a second round of data collection was conducted.

The distribution of the questionnaire was broad and comprehensive, taking into consideration the varying levels of economic development, geographic locations, and the unique characteristics of rural development in different provinces. The questionnaire encompassed 26 provinces spanning eastern, central, western and north-east China, covering over 400 villages and towns for the farmer survey. Moreover, to focus on specific key regions, the questionnaire collection was concentrated in 11 provinces, including Zhejiang, Anhui, and Jiangsu.

Adhering strictly to the principles of stratified sampling, a team of investigators underwent thorough training. Following the selection of research provinces, different counties and districts were chosen based on the economic and social development of each region and their geographic locations. The selection of counties and districts led to the corresponding selection of villages and farmers.

To ensure the thoroughness of the questionnaires, college students and postgraduates from Ningbo University were recruited as data collectors. Each participant in data collection received training. To maintain the quality of the questionnaires, no more than 10 questionnaires were collected per person.

Given the primary focus of this study on the impact of smartphone usage on farmers’ environmental satisfaction, data related to satisfaction with the quality of the rural environment was screened. The data covers various aspects, including basic information about farmers’ families and villages, internet usage, “three wastes” management, waste classification, and environmental satisfaction. After excluding singular or inaccurate values and other cases, a total of 2,632 valid datasets were obtained for the purposes of this study.

### 3.3 Variables

#### 3.3.1 Dependent variable

Dependent variable is rural residents’ environmental satisfaction. Rural environmental satisfaction is an important indicator of wellbeing, representing the sensory evaluation of rural residents regarding environmental quality (15, 16). Typically, environmental satisfaction is measured using the Likert 5-point scoring method (42, 43). In line with previous research experience, this study employs the question, “How satisfied are you with the local environmental quality?” with responses recorded using the Likert 5-point scoring method, ranging from 1 (very dissatisfied) to 5 (very satisfied) (41). This results in an ordered discrete variable, allowing for the establishment of an ordered Probit model to test hypotheses.

#### 3.3.2 Core explanatory variable

##### 3.3.2.1 Smartphone use

Core explanatory variable is smartphone use. Smartphone use is defined as the behavioral patterns and procedural processes wherein individuals leverage smartphones as mobile intelligent terminals to access, operate, and engage with diverse functional applications and contextual activities (44, 45). This includes two aspects: first, the behavioral aspect—i.e., whether an individual uses a smartphone; second, the functional aspect, which involves the scope, frequency, and practical roles of utilizing various mobile application functions (43, 44). Micro-level surveys have commonly used the indicator “whether or not individuals frequently use smartphones to acquire information” (15, 16, 36). This approach is relatively common, validated in existing literature, and has proven effective (Zhang et al., 2018) (15, 16). In alignment with this study’s focus, we choose to measure the degree of smartphone usage in rural areas by asking whether individuals frequently use mobile applications like WeChat and Weibo to acquire information (46). If rural residents frequently use smartphones to access information, it is assigned a value of 1; otherwise, it is assigned a value of 0.

#### 3.3.3 Control variables

In accordance with previous research and the needs of this study, we have selected several control variables. These variables are not the primary focus of this study and are used as control variables to account for factors outside the core explanatory variable that could influence the dependent variable. The selection of control variables is based on two directions. First, we have chosen basic characteristics that may affect individual rural residents’ environmental satisfaction (25, 47, 48), including gender, age, education level, political affiliation, party membership, and annual per capita income (15). Additionally, the installation of public surveillance cameras can significantly affect residents’ environmental satisfaction, so it is included in the control variables. The second direction involves regional variables that could influence smartphone usage (36). Based on geographical divisions into eastern, central, western, and northeastern regions, these are also included in the scope of control variables (25, 49). For detailed variable definitions and descriptive statistics, please refer to Table 1. As shown in Table 1, the smartphone usage rate among rural residents in the sample is 79.7%, and the distribution of various variables is quite broad, aligning with the foundation of the sample analysis, providing a basis for subsequent empirical research.

**Table 1:** Descriptive statistics of variables.

Variable	Definition	Mean	SD	Max	Min
Dependent variable					
Eqs	Assign values from very dissatisfied to very satisfied, in sequence, from 1 to 5	3.424	0.857	1	5
Core explanatory variable					
SU	Do you frequently use mobile applications such as WeChat, Weibo, etc., to obtain information? Assign a value of 1 if yes, and 0 if not	0.797	0.403	0	1
Control variables					
Gender	Female = 0; male = 1	0.524	0.500	0	1
Age	Assign values 1–7 in order from 18 and below to 60 and above.	4.601	1.587	1	7
Education	Assign a value of 1–5 from primary and below to postgraduate and above.	2.498	1.082	1	5
Village cadres	Yes = 1, No = 0	0.0581	0.234	0	1
Party member	Yes = 1, No = 0	0.164	0.370	0	1
Income	Average net household income ranges from less than RMB 5,000 to more than RMB 30,000, with values from 1 to 6 in ascending order.	3.606	1.902	1	6
Security cameras	Are security cameras installed in your village? Yes = 1, No = 0	0.737	0.440	0	1
East	Yes = 1, No = 0	0.557	0.497	0	1
Middle	Yes = 1, No = 0	0.228	0.420	0	1
West	Yes = 1, No = 0	0.206	0.404	0	1
North-east	Yes = 1, No = 0	0.0095	0.0970	0	1

## 4 Empirical study

### 4.1 Baseline regression

Analyzing the results of models (1) to (3) in Table 2, model (1) directly regresses on the core variable. Model (2) controls for variables related to smartphone and individual village usage, while model (3) adds regional control variables. In all cases, the coefficients for the core explanatory variable, smartphone usage, on the assessment of environmental quality satisfaction, are significantly negative, indicating that smartphone usage reduces the individual environmental quality assessment of rural residents.

**Table 2:** Basic regression.

	(1)	(2)	(3)
Variables	Eqs	Eqs	Eqs
SU	−0.161***	−0.129**	−0.123**
	(0.052)	(0.059)	(0.059)
Gender		−0.020	−0.011
		(0.043)	(0.043)
Age		0.048***	0.043**
		(0.018)	(0.018)
Education		−0.006	−0.013
		(0.026)	(0.026)
Village cadres		0.218**	0.221**
		(0.096)	(0.096)
Party member		0.084	0.087
		(0.063)	(0.064)
Income		0.035***	0.021*
		(0.012)	(0.012)
Security cameras		0.245***	0.194***
		(0.049)	(0.050)
East			0.305
			(0.217)
Middle			0.175
			(0.219)
West			0.059
			(0.219)
Observations	2,632	2,632	2,632
Pseudo R-squared	0.00147	0.0130	0.0160
chi^2^(11)	9.655	85.66	105.5

Standard errors are in parentheses; *, ** and *** indicate level of significance at the 10, 5 and 1% level, respectively. The following tables have the same notes and are not labelled as such.

Specifically, considering the results in column (3), the coefficient for smartphone usage is −0.123 (significant at a 5% level), suggesting that rural residents who frequently use smartphones are significantly less satisfied with environmental quality compared to those who do not use smartphones as often. This aligns with theoretical analyses and is generally consistent with findings by Zhang et al. (15).

In consideration of the potential limitations of using ordered probability models, as the coefficients may not accurately reflect the true relationship between variables, marginal effects were computed to provide a more precise analysis. The results are presented in Table 3, showing that, compared to non-smartphone users, smartphone users in rural areas exhibit an increased probability of being ‘Very dissatisfied,’ ‘relatively dissatisfied,’ and ‘general’ by 0.595, 1.75, and 2.45%, respectively. Simultaneously, the probability of being ‘relatively satisfied’ and ‘very satisfied’ decreased by 2.86 and 1.93%. This indicates that the use of smartphones by rural residents does not effectively improve their satisfaction with environmental quality. The welfare effects of smartphone usage on environmental perception by rural residents do not seem to be manifested.

**Table 3:** Marginal effects of variables.

	(1)	(2)	(3)	(4)	(5)
Variables	Margins1	Margins2	Margins3	Margins4	Margins5
					
SU	0.00595**	0.0175**	0.0245**	−0.0286**	−0.0193**
	(0.00293)	(0.00835)	(0.0117)	(0.0136)	(0.00924)
Gender	0.000514	0.00151	0.00212	−0.00247	−0.00167
	(0.00207)	(0.00607)	(0.00851)	(0.00993)	(0.00672)
Age	−0.00210**	−0.00615**	−0.00863**	0.0101**	0.00681**
	(0.000914)	(0.00259)	(0.00362)	(0.00422)	(0.00287)
Education	0.000607	0.00178	0.00250	−0.00291	−0.00197
	(0.00127)	(0.00373)	(0.00522)	(0.00610)	(0.00413)
Village cadres	−0.0107**	−0.0314**	−0.0440**	0.0514**	0.0348**
	(0.00481)	(0.0137)	(0.0191)	(0.0223)	(0.0151)
Party member	−0.00420	−0.0123	−0.0173	0.0202	0.0136
	(0.00311)	(0.00903)	(0.0126)	(0.0147)	(0.00999)
Income	−0.00103*	−0.00301*	−0.00422*	0.00492*	0.00333*
	(0.000591)	(0.00171)	(0.00239)	(0.00278)	(0.00189)
Security cameras	−0.00940***	−0.0276***	−0.0387***	0.0451***	0.0305***
	(0.00267)	(0.00719)	(0.00999)	(0.0116)	(0.00799)
East	−0.0147	−0.0432	−0.0606	0.0707	0.0478
	(0.0106)	(0.0308)	(0.0432)	(0.0503)	(0.0341)
Middle	−0.00845	−0.0248	−0.0348	0.0406	0.0274
	(0.0106)	(0.0310)	(0.0435)	(0.0507)	(0.0344)
West	−0.00287	−0.00842	−0.0118	0.0138	0.00932
	(0.0106)	(0.0311)	(0.0436)	(0.0509)	(0.0344)
Observations	2,632	2,632	2,632	2,632	2,632

Regarding the control variables, Age, Village cadre, Income, and Security cameras yielded positive and significant regression results, while the other variables did not show significance. Among them, Age, Village cadre, Income, and Security cameras have a significant positive impact on rural environmental quality satisfaction. Variables such as Gender, degree, Party membership, and regional variables do not significantly affect environmental quality satisfaction. It is possible that individual characteristics like gender, education level, and party membership do not pose significant barriers to environmental perception, indicating relatively equal conditions in environmental perception among rural residents.

### 4.2 Robustness discussion

To ensure the robustness of the analysis, the study used different regression models for adjustments. Ologit models and Ordinary Least Squares (OLS) models are commonly employed in existing research to mitigate potential methodological biases. The results in Table 4 show that in models 1–4, with or without the addition of control variables, the coefficients for the core explanatory variable remain significantly negative. This confirms that smartphone usage does not enhance environmental quality satisfaction, suggesting the overall model’s robustness.

**Table 4:** Robustness checks 1—alternative specification.

	(1) Ologit	(2) Ologit	(3) Ols	(4) Ols
Variables	Eqs	Eqs	Eqs	Eqs
SU	−0.246***	−0.206**	−0.124***	−0.091**
	(0.091)	(0.103)	(0.041)	(0.046)
Control variables	No	Yes	No	Yes
Regional dummy variables	No	Yes	No	Yes
Observations	2,632	2,632	2,632	2,632
R-squared			0.003	0.033
Pseudo R-squared	0.00110	0.0165		
chi^2^(11)	7.270	108.8		
R^2^_a			0.00303	0.0345
F			8.989	9.550

While controlling for the possibility of endogeneity and potential omitted variables, it is possible that individuals who are more satisfied with their environment may be less inclined to use smartphones, causing reverse causation and model bias. To address this, an alternative variable was considered. In previous studies, “whether to use the internet to obtain information” is often employed as a proxy for internet usage and has been widely used in this field. We believe that using “whether to use the internet to obtain information” as a replacement variable for smartphone usage is reasonable. Smartphone usage is an effective means of accessing information from the internet. Therefore, “whether to use the internet to obtain information” was selected as the core explanatory variable, and the results of the regression can be found in Table 5. It is evident that ICT usage has a significant negative relationship with environmental quality satisfaction (5%). This suggests that substituting the explanatory variable provides a robust explanation, indicating strong robustness in the original research findings.

**Table 5:** Robustness checks 2—replacing the core explanatory variable.

	(1)	(2)	(3)	(4)
Variables	Oprobit	Oprobit	Ologit	Ols
ICT	−0.178***	−0.142**	−0.242**	−0.104**
	(0.053)	(0.061)	(0.107)	(0.048)
Control variables	No	Yes	Yes	Yes
Regional dummy variables	No	Yes	Yes	Yes
Observations	2,632	2,632	2,632	2,632
R-squared				0.039
Pseudo R-squared	0.00169	0.0162	0.0167	
chi^2^(11)	11.15	106.6	109.9	
R^2^_a				0.0348
F				9.632

Considering that the choice of the dependent variable may introduce bias, this study attempted to adjust the dependent variable. Government policy promotion may influence environmental quality satisfaction, and the degree of satisfaction with local environmental governance and environmental protection propaganda is an alternative indicator. Therefore, using “your degree of satisfaction with local environmental governance and environmental protection propaganda” and assigning values ranging from ‘Very dissatisfied’ to ‘Very satisfied’ from 1 to 5, the regression results are presented in Table 6. It is clear that smartphone usage has a significantly negative relationship with satisfaction in environmental propaganda quality (5%), indicating the robustness of the replacement explanatory variable and the strength of the original research findings.

**Table 6:** Robustness checks 3—replacing the dependent variable.

	(1)	(2)	(3)	(4)
Variables	Oprobit	Oprobit	Ologit	Ols
SU	−0.103**	−0.114**	−0.185**	−0.090**
	(0.052)	(0.052)	(0.091)	(0.042)
Regional dummy variables	No	Yes	Yes	Yes
Observations	2,632	2,632	2,632	2,632
R-squared				0.017
Pseudo R-squared	0.000604	0.00698	0.00702	
chi^2^(11)	3.984	46.00	46.32	
R^2^_a				0.0158
F				11.53

To further address robustness, this study incorporates control variables into the analysis. Firstly, given that China was affected by the COVID-19 pandemic both before and after data collection, this extraordinary event may have exerted an impact on residents’ environmental perception. To account for such an impact, the COVID-19 pandemic is included as a control variable in the dataset for analysis. Specifically, the number of confirmed COVID-19 cases and COVID-19-related deaths across China’s provincial-level administrative regions as of December 31, 2020, are used as measurement indicators in the regression. Results presented in Table 7 (1)–(2) show that after incorporating the COVID-19 impact as a control variable, the negative impact of smartphone use on the perceived evaluation of environmental quality remains statistically significant. This validates the significance of the baseline regression results. Secondly, objective environmental conditions at the village level may influence residents’ environmental perception. In China, these village-level objective environmental conditions are strongly associated with government governance (15); thus, the distance from villages or individuals to the local government can serve as an indicator of objective environmental conditions. Specifically, the distance to the local town government is used as a proxy variable, which is incorporated into the regression as a control variable. Results in Table 7 (3) demonstrate that after including the village-level environmental factor as a control variable, the negative impact of smartphone use on the perceived evaluation of environmental quality remains statistically significant. This further confirms the significance of the baseline regression results.

**Table 7:** Robustness checks 4—incorporating control variables.

	(1)	(2)	(3)
Variables	Oprobit	Oprobit	Oprobit
SU	−0.116**	−0.116**	−0.123**
	(0.059)	(0.059)	(0.059)
Control variables	Yes	Yes	Yes
Observations	2,632	2,632	2,632
Pseudo R-squared	0.0170	0.0171	0.0162
chi^2^(12)	111.7	112.3	106.8

### 4.3 Heterogeneity analysis

Firstly, geographical heterogeneity was considered. Due to China’s vast territory and diverse population, there are apparent differences in internet use and living environments among individuals. To analyze the potential impact of regional differences on the results, the data from four major regions (East, Central, West, and Northeast) were used for ordered probit regression, with the results shown in Table 8 (1) to (4). The results indicate that East and Central regions have a significantly negative relationship, while the West and Northeast regions have a non-significant positive relationship. This implies that the impact of digital development on the environmental quality satisfaction of rural residents varies significantly across different regions. This could be attributed to higher internet penetration rates in East and Central regions, with rural residents having higher expectations for environmental governance, potentially resulting in lower satisfaction when they acquire more environmental information. In contrast, the impact of the internet is less pronounced in the West and Northeast, reducing the comparative effect and leaving satisfaction relatively unaffected.

**Table 8:** Regional heterogeneity.

	(1)	(2)	(3)	(4)
Variables	East	Middle	West	Northeast
SU	−0.191**	−0.215*	0.117	1.467
	(0.081)	(0.124)	(0.122)	(1.147)
Control variables	Yes	Yes	Yes	Yes
Observations	1,465	600	542	25
Pseudo R-squared	0.00677	0.0295	0.00830	0.336
chi^2^(11)	24.73	44.15	10.95	19.17

Secondly, analysis of villager identity heterogeneity was performed. In China, rural communities are characterized by strong social ties, and individuals with different political identities have varying abilities to access information and resources, which may lead to different environmental quality satisfaction levels. To explore this potential heterogeneity, a consistency discussion was conducted for village cadre and non-village cadre, as well as party members and non-party members, as shown in Table 9. The results reveal that data regressions for village cadre and party members are not significant, while the data regressions for ordinary villagers show a significantly negative relationship. This suggests that the impact of smartphone usage on the environmental quality satisfaction of rural residents varies significantly depending on whether they are village cadre, party members, or ordinary villagers. This could be due to differences in their perspectives and views. Village cadres and party members have administrative and responsive roles, while villagers have governance, rights protection, and non-participation characteristics. Village cadres and party members are more likely to respond to policies, reflecting policy-oriented responses. In contrast, villagers’ rights protection is more likely to result in dissatisfaction with environmental issues, thereby reflecting negative satisfaction.

**Table 9:** Political identity heterogeneity among rural residents.

	(1)	(2)	(3)	(4)
Variables	Village cadre	Non-village cadre	Party member	Non-party member
SU	0.136	−0.135**	−0.145	−0.120*
	(0.287)	(0.060)	(0.161)	(0.063)
Control variables	Yes	Yes	Yes	Yes
Regional dummy variables	Yes	Yes	Yes	Yes
Observations	153	2,479	431	2,201
Pseudo R-squared	0.0328	0.0152	0.0192	0.0162
chi^2^(11)	11.40	94.38	20.46	89.46

## 5 Further exploration: mechanisms analysis

Based on the empirical analysis discussed earlier, it is evident that smartphone usage has a negative impact on the environmental quality satisfaction of rural residents. To gain a deeper understanding of the mechanisms underlying this impact, we can explore how digitization affects rural residents’ satisfaction from two perspectives.

### 5.1 Substitution of information channels

The core function of smartphone use is information sharing and transmission. As one of the primary channels for contemporary citizens to access information, the internet significantly increases the supply of information, reduces potential risks arising from information asymmetry, and intensifies social comparisons. As depicted in Table 10, smartphone usage has a significant negative impact on traditional information sources such as television. These negative relationships are significant at the 1% level. This implies that the widespread use of smartphones significantly reduces the likelihood of using traditional information sources. Rural residents who frequently exchange information via the internet are more likely to have a negative perception of their environment. Furthermore, smartphone usage has also increased the proportion of people using WeChat group discussion and enjoying online public services. For instance, Model (3) and (6) use “whether they frequently enjoy online public educational services” as a mediating variable to analyze its impact on rural residents’ environmental satisfaction. The results consistently demonstrate a significant positive effect, regardless of whether other variables are controlled or different regression models are employed. This implies that rural residents who frequently receive public educational services online are more likely to have a positive perception of their environment.

**Table 10:** Substitution of information channels.

	(1)	(2)	(3)	(4)	(5)	(6)
Variables	Television	WeChat group discussion	Online education services	Eqs	Eqs	Eqs
SU	−0.364***	0.131***	0.293***	−0.113*	−0.101**	−0.108**
	(0.077)	(0.019)	(0.022)	(0.059)	(0.046)	(0.046)
Television				0.089*		
				(0.046)		
WeChat group discussion					0.151***	
		.			(0.043)	
Online education services						0.139***
						(0.038)
Control variables	Yes	Yes	Yes	Yes	Yes	Yes
Regional dummy variables	Yes	Yes	Yes	Yes	Yes	Yes
Observations	2,632	2,632	2,632	2,632	2,632	2,632
Pseudo R-squared	0.0264	0.018	0.063	0.0166	0.043	0.043
chi^2^(11)	85.90	49.29	176.2	109.3	9.808	9.924

From a media studies perspective, after analyzing sentiment through word frequency in the wake of major events, it is observed that negative sentiment (sadness and anger) on the internet is more prevalent than neutral or positive sentiment (19, 42). This indicates that individuals who are frequently exposed to online information are more likely to internalize negative emotions from the internet. Faced with negative information about environmental quality online, they are more inclined to be dissatisfied with their current environmental conditions. Moreover, for farmers living in areas with severe environmental pollution, internet usage might intensify their comparison between their local environmental quality and that of regions with better environmental conditions, leading to reduced satisfaction with their current environment. Research suggests that the influence of the internet on rural residents’ satisfaction with local environmental quality is more substantial compared to urban residents. Therefore, by expanding information channels, the internet enables rural residents to broaden their cognitive horizons, exposing them to disparities between regions with better environmental quality and their own, ultimately fostering negative emotions and dissatisfaction with their current environment.

### 5.2 Transformation of social capital

In addition to broadening the cognitive horizons of rural residents, the internet has led to significant changes in their social capital structure. Traditionally, social capital in Chinese rural areas was concentrated among political elites, and ordinary farmers needed to access political status through offline channels, such as participating in rural elections (32). With the widespread use and development of the internet, residents’ access to social capital has significantly improved.

First, after using smartphones, traditional offline political participation, such as participating in village elections or voting, is less likely, and online channels are becoming substitutes. The analysis of 2,632 survey responses in this study shows that villagers who frequently use smartphones to obtain information have a higher participation rate in public affairs compared to non-users. They also participate digitally at nearly three times the rate using methods such as online platforms for village management (apps, WeChat official accounts, websites), and digital voting. Model (1) and (4) in Table 11 confirm that smartphone usage significantly reduces the proportion of rural residents participating in offline village-level elections and voting, forming a mechanism with a negative impact on environmental quality perception. Model (2) and (5) in Table 11 illustrate that smartphone usage significantly enhances online means of participating in rural governance, such as village management platforms, creating a mechanism for the positive enhancement of environmental quality assessment. This suggests that smartphone usage shifts residents from offline to online forms of participation in rural governance, reducing the effectiveness of offline channels on environmental quality perception.

**Table 11:** Transformation of social capital.

	(1)	(2)	(3)	(4)	(5)	(6)
Variables	Offline discussion or voting	Village affairs management platform	Network sharing information	Eqs	Eqs	Eqs
SU	−0.062***	0.050***	0.920***	−0.092**	−0.092**	−0.126***
	(0.024)	(0.013)	(0.051)	(0.046)	(0.046)	(0.047)
Offline discussion or voting				0.125***		
				(0.035)		
Village affairs management platform					0.226***	
		.			(0.063)	
Network sharing information						0.057***
						(0.016)
Control variables	Yes	Yes	Yes	Yes	Yes	Yes
Regional dummy variables	Yes	Yes	Yes	Yes	Yes	Yes
Observations	2,632	2,632	2,632	2,632	2,632	2,632
Pseudo R-squared	0.003	0.006	0.110	0.043	0.043	0.043
chi^2^(11)	6.732	14.98	326.3	9.871	9.878	9.815

Second, after using smartphones, people find it easier to access information, communicate with others, and share information, thereby expanding their online social capital channels. Frequent online communication can help individuals gain a more accurate understanding of their surroundings. Model (3) and (6) confirm that smartphone usage significantly enhances rural residents’ means of sharing their experiences, creating a mechanism for positively improving environmental quality assessment. This indicates that residents reconstruct their online social capital structure by sharing their experiences, contributing to a heightened perception of environmental quality.

In summary, after smartphone usage, rural residents, on the whole, exhibit a negative perception of environmental quality. This could be due to the widespread use of the smartphone, which nurtures a sense of entitlement among farmers and may potentially influence real-world opinion. The smartphone’s characteristics, including free expression, interpersonal interaction, and equal dialogue, easily cultivate qualities of citizenship such as equality, independence, self-esteem, and participation, further raising awareness of rights. Information dissemination becomes more transparent and rapid, enabling individuals to scrutinize and expose instances of injustice in society. The smartphone also provides a platform for those whose rights are violated to organize and initiate actions for their rights. Online activities such as petitions, gatherings, and signatures enable more people to participate in rights protection actions, amplifying their voices and pressure. Against the backdrop of smartphone usage proliferation, rural residents’ awareness of environmental protection has been steadily rising, leading to increased smartphone’s negative effects on their environmental quality satisfaction.

The mechanism analysis, notably, confirms that despite the inundation of negative information online and its potential negative impact on residents’ satisfaction levels, normal online sharing activities indicate that rural residents are stepping out of their own world to engage in online communication, effectively expanding their online social capital. Additionally, active government efforts to promote rural digitization have improved rural digital governance (e.g., voting activities) and public digital services (e.g., online education). These efforts are vital in guiding residents towards intrinsic wellbeing, supported by the empirical evidence presented in this study.

## 6 Conclusion and policy recommendations

The conclusions drawn from this study are as follows: first, based on the baseline regression results, it is evident that smartphone usage significantly negatively impacts the environmental quality satisfaction of rural residents. The ordered probit model results confirm that, without controlling for other variables, after adding control variables, and after including regional factors, smartphone usage significantly negatively affects the environmental quality satisfaction of rural residents. Multiple robustness checks using different regression models (OLS, binary probit, binary logit) and variations of core variables validate the stability of this result.

Second, from the heterogeneity analysis, it becomes apparent that there are significant regional differences in the impact of digital development on rural residents’ quality satisfaction. In the eastern and central regions, smartphone usage significantly negatively affects environmental quality satisfaction, while in the western and northeastern regions, there is no significant impact. The differences in villagers’ status also significantly influence the environmental quality satisfaction of rural residents. The use of smartphones does not significantly affect the satisfaction of residents with roles such as village cadres and party members, but it significantly negatively affects the satisfaction of ordinary villagers without such positions.

Third, from the mechanism analysis, it can be concluded that digital development influences environmental quality satisfaction through the improvement of rural residents’ information channel substitution and the transformation of social capital. Concerning the substitution of information channels, smartphone usage becomes an alternative to traditional information sources. This indicates that the widespread use of smartphones significantly reduces the probability of using traditional information channels (television, landline phones, radio, newspapers, friends, village committees). In terms of the transformation of social capital, after smartphone usage, there is a decreased likelihood of participating in traditional offline political activities, replaced by online channels. People find it easier to access information, communicate with others, share information, and the proportion of those enjoying online public services also increases. Smartphone usage squeezes the space for rural residents to participate in offline governance activities, thus limiting residents’ environmental quality perception to some extent. Simultaneously, normal online sharing activities among rural residents indicate that they are engaging in online communication, effectively expanding their online social capital, which is an effective means of improving environmental wellbeing.

As indicated by the findings of this paper, the technological development of the smartphone usage has a negative impact on some aspects of residents’ environmental quality satisfaction and wellbeing. Based on these conclusions, the following policy recommendations can be proposed:

Firstly, in the digital age, policymakers should consider the impact of technological factors on social development when formulating specific policies and conducting risk prevention. Given that digital development currently has a negative impact on the environmental quality perception of rural residents, the government should consider the consequences of online public opinion and digital communication when developing specific policies for rural environmental remediation.

Secondly, in village environmental remediation, attention should be given to regional differences between the eastern and western regions and the identity of villagers. Further efforts should be made to promote rural environmental governance in the central and western regions. Policies adapted to local customs, economic development, and the level of digital development should be implemented to reduce regional disparities. It is essential to enable village cadres and party members to play a leading role while effectively supervising and reviewing the results of rural environmental governance, preventing any embellishment of these outcomes.

Thirdly, the smartphone usage can serve as a significant force in the government’s environmental actions in the digital era. The government should make good use of smartphone to promote grassroots work. As found in this study, the smartphone’s widespread use expands rural residents’ awareness of environmental issues, motivating them to take action to protect their rights based on this information. More frequent internet usage enables rural residents to utilize online channels and information provided by the internet to coordinate their dissatisfaction with the authorities, rather than engaging in impulsive actions. Actively promoting digital rural development by improving digital governance in rural areas (e.g., digital voting activities) and enhancing digital public services (e.g., online education) is a crucial path to guiding residents towards intrinsic wellbeing. The research presented in this paper provides empirical evidence to support this approach.

The proliferation of smartphones and the development of internet digitization are reshaping the ways of thinking and behavioral choices of rural residents, affecting various aspects of rural life. Environmental quality perception is a vital component of residents’ wellbeing and an important means of improving the welfare of rural residents. This paper utilized data from the 2020 survey on modern rural governance to explore the impact of smartphone usage on the environmental quality satisfaction of rural residents. The analysis focused on the characteristics of the internet as a medium and examined the mechanisms through which digital development influenced changes in rural residents’ social capital. This paper innovatively explored the mechanisms underlying the enhancement of rural residents’ wellbeing and satisfaction. Naturally, this paper also has certain limitations. Firstly, it uses cross-sectional data to study the impact of smartphone usage on the environmental quality satisfaction of rural residents, while panel data may better address endogeneity issues. Secondly, there is a lack of more precise quantification in the description and measurement of smartphone usage development. Also, for instance, there are shortcomings in the selection of instrumental variables and the practical application of mechanisms. In future research, it will focus on exploring the impacts of different application functions of smartphones on various dimensions of residents’ environmental perceptions, striving to achieve a more refined measurement.

## Data Availability

The raw data supporting the conclusions of this article will be made available by the authors, without undue reservation.

## Footnotes

1 Please visit: https://news.qq.com/rain/a/20250722A02W3I00#:~:text (accessed on 13 November 2025).

2 Please visit: https://finance.sina.com.cn/tech/roll/2025-07-31/doc-infikkxp2720091.shtml (accessed on 13 November 2025).
